# Early pathogenic event of Alzheimer’s disease documented in iPSCs from patients with PSEN1 mutations

**DOI:** 10.18632/oncotarget.13776

**Published:** 2016-12-02

**Authors:** Juan Yang, Hanzhi Zhao, Yu Ma, Guilai Shi, Jian Song, Yu Tang, Song Li, Ting Li, Nan Liu, Fan Tang, Junjie Gu, Lingling Zhang, Zhuohua Zhang, Xiaohui Zhang, Ying Jin, Weidong Le

**Affiliations:** ^1^ Key Laboratory of Stem Cell Biology, CAS Center for Excellence in Molecular Cell Science, Institute of Health Sciences, Shanghai Institutes for Biological Sciences, Chinese Academy of Sciences/Shanghai JiaoTong University School of Medicine, Shanghai 200031, China; ^2^ Shanghai Stem Cell Institute, Shanghai JiaoTong University School of Medicine, Shanghai 200025, China; ^3^ Center for Clinical Research on Neurological Diseases, the First Affiliated Hospital, Dalian Medical University, Dalian 116021, China; ^4^ Institute of Neuroscience, Shanghai Institutes of Biological Sciences, Chinese Academy of Sciences, Shanghai 200031, China; ^5^ Institute of Precision Medicine, The Xiangya Hospital, State Key Laboratory of Medical Genetics, Xiangya Medical School, Central South University, Changsha 410078, China; ^6^ Institute of Neurology, Ruijin Hospital, Shanghai JiaoTong University School of Medicine, Shanghai 200025, China; ^7^ Collaborative Innovation Center for Brain Science, the First Affiliated Hospital, Dalian Medical University, Dalian 116011, China

**Keywords:** Alzheimer's disease, apoptosis, neural progenitor cells, premature neuronal differentiation, induced pluripotent stem cells

## Abstract

Alzheimer's disease (AD) is the most common age-related dementia characterized by progressive neuronal loss. However, the molecular mechanisms for the neuronal loss is still debated. Here, we used induced pluripotent stem cells (iPSCs) derived from somatic cells of familial AD patients carrying *PSEN1* mutations to study the early pathogenic event of AD. We found that premature neuronal differentiation with decreased proliferation and increased apoptosis occured in AD-iPSC-derived neural progenitor cells (AD-NPCs) once neuronal differentiation was initiated, together with higher levels of Aβ_42_ and phosphorylated tau. Premature neuronal differentiation in AD-NPCs was caused by *PSEN1* mutations and might be correlated to multiple dysregulated processes including but not limited to Wnt-Notch pathway. Our study documented previously unappreciated early NPC dysfunction in AD-NPCs, providing valuable new insights into the early mechanisms underlying AD pathogenesis.

## INTRODUCTION

Alzheimer's disease (AD) is a progressive and irreversible neurodegenerative disorder of the central nervous system and presents the most common form of age-associated dementia. Mutations in amyloid precursor protein (*APP*), presenilin-1 (*PSEN1*) or presenilin-2 (*PSEN2*) genes cause familial AD (FAD) in an autosomal dominant manner [1]. Specially, mutations in *PSEN1* are identified to be the most common cause of FAD with complete penetrance and tend to manifest the early onset of the disease [2, 3]. Similar brain neuropathology is observed in both sporadic and familial forms of the disease, suggesting that common cellular mechanisms may be responsible for the development of AD [4]. Therefore, studies in FAD patients with known genetic defects have greatly contributed to our understanding of AD at molecular level.

AD has clinical features of progressive memory decline and cognitive disturbance, and neuropathological hallmarks amyloid plaques and neurofibrillary tangles in the postmortem brain [5, 6]. Amyloid plaques are extracellular aggregates of β-amyloid (Aβ), whereas neurofibrillary tangles consist of cytoplasmic filaments of hyper-phosphorylated tau (p-tau) proteins. Aβ is produced from APP through the sequential proteolytic action of β- and γ-secretases. PSEN1 is considered to be critical for the γ-secretase complex, acting as the catalytic component of this proteolytic enzyme [7].

Although the pathological features of AD have been extensively investigated, the relationship between known pathological features and disease progression remains unclear. In APP-transgenic mice, specific spatial learning impairment is evident in young mice and becomes severe with age without the formation of plaques [8]. Moreover, it is reported that neuronal loss, but not the plaque and tangle, in AD patients’ brain is closely associated with the cognitive decline [9]. Indeed, it is reported that pathological and biochemical changes of AD in cerebrospinal fluid, brain metabolism, brain amyloid deposition, as well as cognitive impairment may have existed for more than 20 years before the recognized symptom onset in FAD [10]. Therefore, it is urgently needed to identify the early events of AD pathogenesis and to develop effective strategies for disease-modifying therapy at the early stage of AD.

The limited experimental access to AD-affected human brain tissue has severely impeded the elucidating of early molecular mechanisms underlying AD development. Generation of induced pluripotent stem cells (iPSCs) by over-expression of defined transcription factors in somatic cells, particularly in those from patients, presents an attractive and promising approach to model early stages of AD *in vitro* and to screen novel biomarkers as well as therapeutic medicines [11–14]. To date, several research groups have independently reported that AD patient-specific iPSC-derived neurons and glia recapitulate multiple features of AD pathological events, offering experimental evidence of utilizing patient-specific iPSCs to model AD and reevaluate the current hypothesis of AD pathogenesis [15–22]. Their findings also suggest that iPSCs from large numbers of AD patients are needed for the understanding of AD etiology comprehensively due to the heterogeneity of familial and sporadic AD [23].

In an attempt to investigate whether there would be alterations in the early stage of neural differentiation from AD-iPSCs, we generated four iPSC lines from fibroblasts of two FAD patients and differentiated them into neural progenitor cells (NPCs). Interestingly, when neuronal differentiation was initiated, these AD-iPSC-derived NPCs (AD-NPCs) displayed premature neuronal differentiation and enhanced apoptosis as well as reduced proliferation, which leads to a decreased NPCs number during differentiation and might be relevant to the neuronal loss of AD. Therefore, this study documents previously unappreciated early neural dysfunctions in NPCs from AD-iPSCs, providing new cues for elucidating early molecular mechanisms underlying AD development.

## RESULTS

### Generation and characterization of iPSC lines from fibroblasts of FAD patients with PSEN1 mutations

Four iPSC lines were generated from skin fibroblasts of two FAD patients with *PSEN1* mutations, including one line from a patient bearing a mutation of three-nucleoside-deletion (heterozygous S169del, termed A16-iPSC-1) and three lines from another patient carrying a missense mutation (heterozygous A246E, termed A15-iPSC-2, -3, and -4, respectively) (Supplementary Figure 1a and 1b). Two previously established iPSC lines derived from fibroblasts of an unrelated normal individual were used as controls (termed N-iPSC-1 and -2) [24]. All iPSCs were generated through the infection of fibroblasts with retroviruses encoding *OCT4*, *SOX2*, *KLF4* and *C-MYC*, displaying typical features of human pluripotent stem cells (Supplementary Figure 1c-1f). Fully reprogramming in the established iPSC lines was further validated at a global transcriptional level through mRNA microarray analyses (Supplementary Figure 1g).

The differentiation capacity of the established AD- and N-iPSCs was examined through embryoid bodies (EBs) (Supplementary Figure 1h and 1i) and teratoma formation assays (Supplementary Figure 1j and 1k). Moreover, karyotype analysis showed that both A16-iPSCs and A15-iPSCs bore a karyotype of 46,XX (Supplementary Figure 1l), however all the A15-iPSCs carried a translocation between chromosome 1 and 9,t(1;9)(p32;q11). The short tandem repeat analysis verified the distinct identity of iPSC lines established in this study (Supplementary Table 1). Altogether, our results indicated that the iPSCs generated in this study behaved as typical human pluripotent stem cells with potential to differentiate into cell types of all three germ layers both *in vitro* and *in vivo*, without detectable differences in the developmental potential between AD- and N-iPSCs

### AD-iPSCs differentiate into NPCs normally but premature neuronal differentiation occurs during differentiation of AD-NPCs

**Figure 1:**
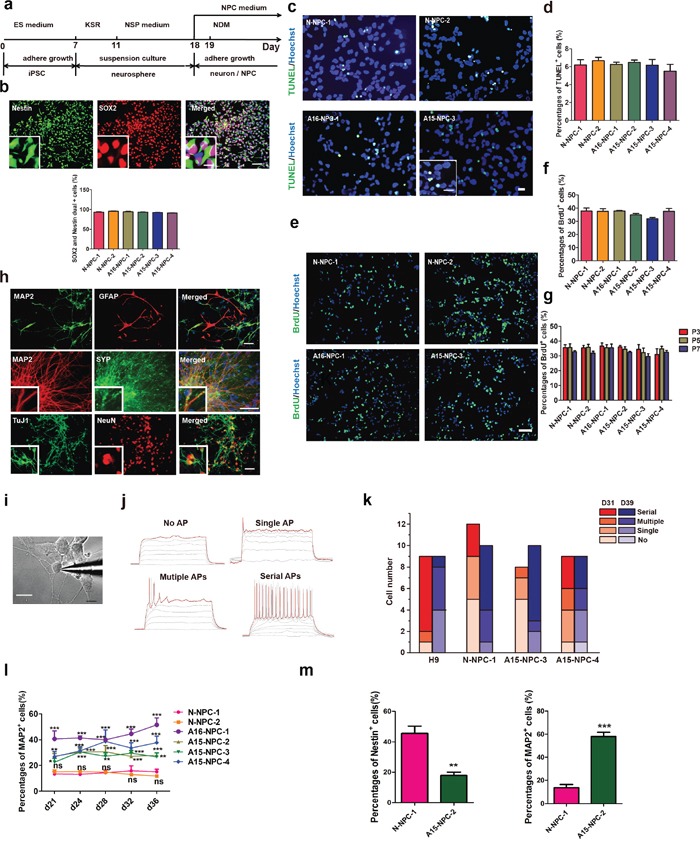
Neural differentiation and characterization of iPSCs **a.** Schematic diagram of our neural differentiation protocol. **b.** Immunofluorescence staining and quantification of neural progenitor cells (NPCs) derived from neurospheres with antibodies against Nestin (green) and SOX2 (red). The inset shows a higher magnification view. Scale bar, 50 μm. The error bars indicate SEM. n=3. **c** and **d.** Immunofluorescence staining (c) and quantification (d) of apoptosis of NPCs by TUNEL staining. Scale bar, 50 μm. The error bars indicate SEM. n=3. **e** to **g.** Immunofluorescence staining (e) and quantification (f) of proliferation abilities of NPCs as well as the percentages of BrdU+ NPCs at different passages (g). The error bars indicate SEM. n=3. **h.** Representative immunofluorescence images of glia (GFAP+, red) and neurons (MAP2+, green) derived from NPCs as well as other markers of neurons including TuJ1, (green), NeuN (red) and SYP (green). Scale bars, 50 μm. **i.** Bright field images showing whole-cell recording on a cultured iPSC under DIC microscope. Scale bars, 10 μm. **j.** The classification of firing capability: No AP, no action potential was observed in all voltage traces; Single AP, only one AP evoked at the beginning of suprathreshold current injection; Multiple APs, multiple APs evoked in the early time of the injection of step currents; Serial APs, serial APs evoked along whole period of step current injection. **k.** Statistic analysis of percentages of spiking cells recorded from neurons derived from each cell line. D31: N-NPC-1, n=12; A15-NPC-3, -4, n=8. D39: N-NPC-1, n=10; A15-NPC-3, n=10; A15-NPC-4, n=9. **l.** Neuronal differentiation efficiencies of each cell line by RA-induced protocol were determined by the percentage of MAP2+ cells on different days of differentiation and compared by Two-way ANOVA. The error bars indicate SEM. ns, not significant;*, p< 0.05; **, p<0.01; ***, p<0.001; n> or = 5. **m.** The percentages of MAP2+ cells or Nestin+ cells on day 28 of monolayer neuronal differentiation. The error bars indicate SEM.**, p<0.01; ***, p<0.001; n=3.

We induced iPSCs to differentiate towards NPCs by retinoic acid (RA) treatment and neurosphere formation based on a published protocol (Figure 1a) [25]. After 18 days of differentiation, both N-iPSCs and AD-iPSCs displayed typical morphological feature of NPCs and more than 90% of the cells expressed NPC markers (Nestin and SOX2) (Figure 1b). In addition, we found that the apoptosis and proliferation capacity of NPCs was indistinguishable between N-iPSC-derived NPCs (N-NPCs) and AD-NPCs when cultured in the NPC medium containing basic fibroblast growth factor (bFGF) (Figure 1c-1g). Therefore, our results showed that mutations in *PSEN1* did not disrupt the normal survival and proliferation of AD-NPCs.

We then induced AD-NPCs and N-NPCs into neurons and examined whether AD-NPC-derived neurons would exhibit normal features of neurons at molecular and functional levels. NPCs from both AD-iPSCs and N-iPSCs could spontaneously differentiate into glia or neurons of various types after withdrawal of bFGF from the NPC medium (Figure 1h and Supplementary Figure 2), suggesting that NPCs of both types had a potential to differentiate into major neural cell types. Moreover, there was no observable difference in electrophysiology properties among the neurons from AD-NPCs and N-NPCs (Figure 1i-1k).

**Figure 2:**
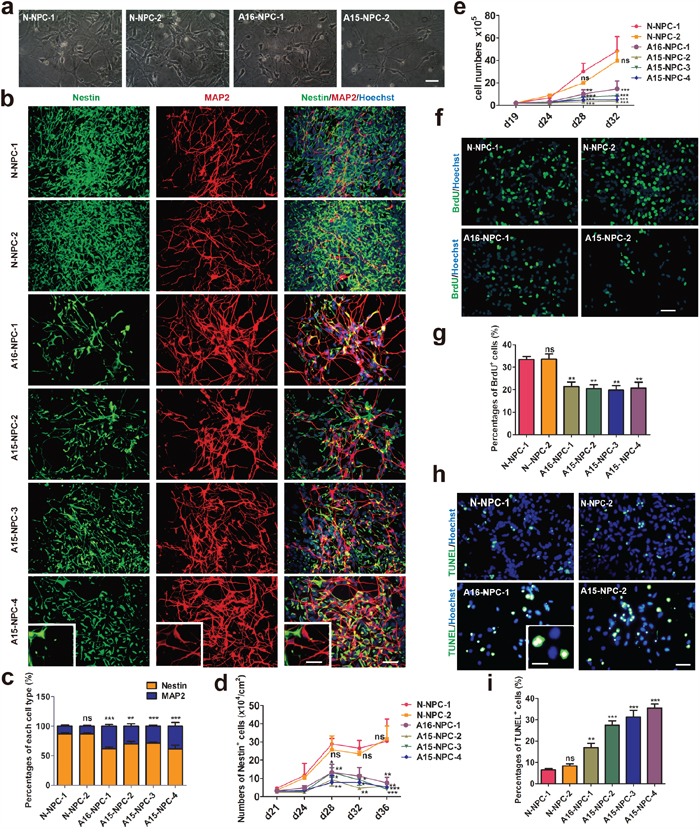
Premature neuronal differentiation occurs in differentiating AD-NPCs **a.** Representative images of neural cells at day 28 of differentiation. Scale bar, 50 μm. **b.** Immunofluorescence staining images of each cell line at day 28 of differentiation with MAP2 and Nestin antibodies. Scale bar, 50 μm. The inset shows a higher magnification view. Scale bars, 20 μm. **c.** Statistic analyses of percentages of Nestin+ and MAP2+ cells derived from each cell line after differentiation for 28 days. The percentage of MAP2+ cells of each cell line was compared with that of N-NPC-1 line by the Student's t-test (n > or = 5; ns, not significant, **p<0.01, ***p<0.001). **d.** The numbers of NPCs per cm2 of each cell line at different days of differentiation were compared by two-way ANOVA (n> or = 5; mean ± SEM; ns, not significant, *p<0.05, **p<0.01, ***p<0.001). **e.** Cell numbers of various cell line duringdifferentiation in each well of 6-well plates were counted at different days of differentiation (n=3; mean ± SEM; ns, not significant, *p<0.05, **p<0.01, ***p<0.001). **f** and **g.** Immunofluorescence staining (f) and quantification (g) of the proliferation ability of neural cells by BrdU incorporation assays on day 28 of differentiation. Scale bar, 50 μm (n = 4; mean ± SEM; ns, not significant, **p<0.01). **h** and **i.** Immunofluorescence staining (h) and quantification (i) of apoptotic neural cells by TUNEL staining assays on day 28 of differentiation. Scale bar, 50 μm (n = or > 3; mean ± SEM; ns, not significant, **p<0.01, ***p<0.001).

However, we noticed different cellular morphologies and densities between AD-NPC and N-NPC cultures immediately after the initiation of neuronal differentiation (Figure 2a). Many cells of AD-NPCs exhibited the typical neuron morphology with long neurite outgrowth, whereas the majority of cells of N-NPCs maintained a typical NPC morphology (Figure 2a). To assess the percentages of NPCs and neurons in the differentiation culture, we carried out immunofluorescence staining using antibodies against Nestin and MAP2 at day 28 of differentiation. Significantly lower percentages of Nestin^+^ NPCs but higher percentages of MAP2^+^ neurons were found in AD-NPCs, compared to those in N-NPCs (Figure 2b and 2c). Similar results were obtained from differentiating cells at other days examined (Figure 2d and Figure 1l). Moreover, the number of Nestin^+^ cells in the AD-NPCs culture was significantly less than that in N-NPCs during differentiation from day 28 to 36 (Figure 2d). In addition, we detected higher percentages of neurons and lower percentages of NPCs in AD-NPCs generated through a widely used SMAD inhibition neural differentiation protocol [26, 27], suggesting that the phenotype of premature neuronal differentiation observed during differentiation of AD-NPCs is not dependent on the NPCs generation strategy (Figure 1m).

Accompanied with the premature neuronal differentiation, the cell growth rate of AD-NPCs was significantly lower than that of N-NPCs during this process (Figure 2e). Moreover, significantly lower percentage of BrdU^+^ proliferative cells was detected in differentiating AD-NPCs compared to those in control cells on day 28 of differentiation (Figure 2f and 2g), in accordance with lower percentage of Nestin^+^ NPCs and less number of Nestin^+^ cells in AD-NPCs during differentiation. Furthermore, higher percentage of TUNEL^+^ apoptotic cells was observed in differentiating AD-NPCs on day 28 (Figure 2h and 2i).

### Typical AD pathological changes and degenerating neurons are found during neuronal differentiation of AD-NPCs

**Figure 3:**
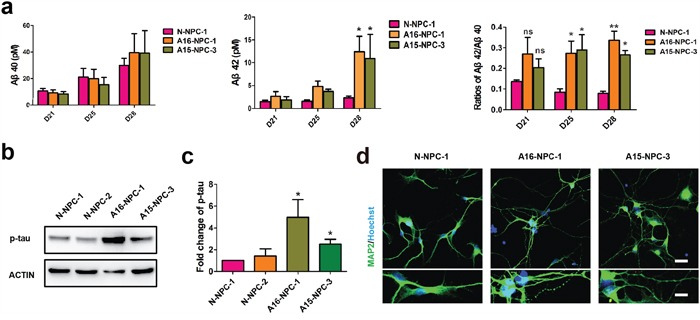
Typical AD pathological changes and degenerating neurons are observed in neuronal differentiation of AD-NPCs **a.** ELISA of Aβ40 and Aβ42 during neuronal differentiation (n = 3; mean ± SEM; ns, not significant, *p<0.05, **p<0.01). **b** and **c.** Western blotting (b) and quantification (c) of p-tau protein levels in differentiating NPCs at day 28 (n = 4; mean ± SEM;*p< 0.05). **d.** Neurite fragmentation in neurons from AD-NPCs, scale bars, 50 μm. Lower panels are higher magnification views of neurons. Scale bar of upper panels, 200 μm; scale bar of lower panels, 100 μm.

We found that the level of secreted Aβ_42_ was significantly higher in both A15 and A16 AD-NPCs differentiation culture than that in N-NPCs differentiation culture on day 28, while AD-NPCs differentiation culture had a significantly elevated ratio of Aβ_42_/Aβ_40_ than N-NPCs differentiation culture at day 25 and day 28 of neuronal differentiation (Figure 3a). Moreover, protein levels of p-tau were higher in both A15 and A16 differentiating AD-NPCs compared to those of differentiating N-NPCs on day 28 (Figure 3b and 3c). Considering that the premature neuronal differentiation was evident in AD-NPCs at as early as differentiation day 21, it was possible that the premature neuronal differentiation occurred prior to the alterations of Aβ_42_/Aβ_40_ ratio and p-tau levels.

Interestingly, we observed the neurite fragmentation phenotype on neurons derived from AD-NPCs frequently, whereas this phenotype was rarely detected in the neurons generated from N-NPCs (Figure 3d). Thus, AD patient iPSC-derived neurons could model the disease not only at a molecular level but also morphologically.

### Over-expression of mutated PSEN1 in N-iPSCs results in premature neuronal differentiation

**Figure 4:**
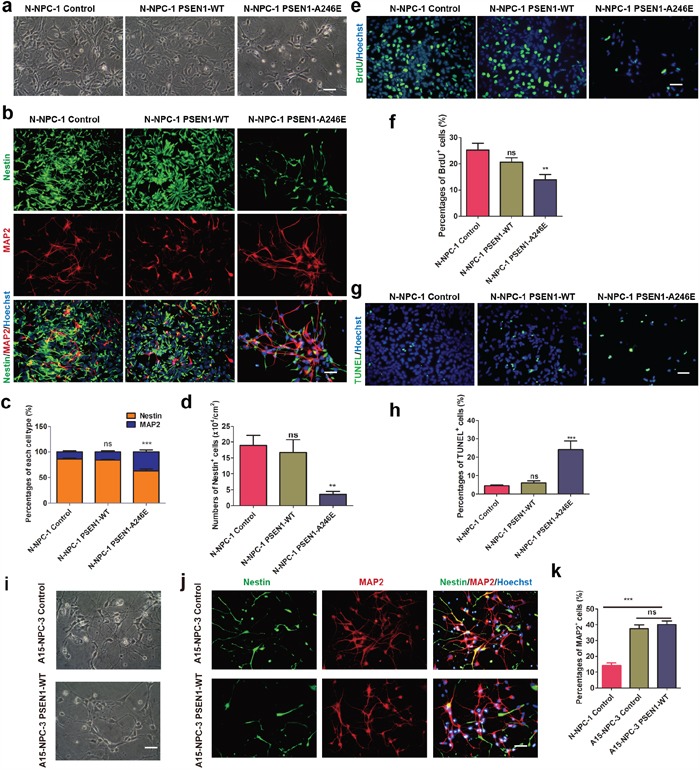
PSEN1-A246E is responsible for the abnormal neuronal differentiation phenotype **a.** Representative bright field images at day 28 of differentiation form NPCs of N-iPSC-1 line transduced with the vector, PSEN1-WT and PSEN1-A246E, respectively. Scale bar, 50 μm. **b.** Immunofluorescence staining images of each cell line at day 28 of differentiation with antibodies against MAP2 and Nestin. Scale bar, 50 μm. **c** and **d.** Quantitative analysis of neuronal differentiation efficiencies (c) and numbers of NPCs per cm2 (d) of each cell line on day 28 (n = 5; mean ± SEM; ns, not significant, **p<0.01, ***p<0.001). **e** and **f.** Immunofluorescence staining of BrdU (e) and quantification of BrdU+ cells (f) of each cell line during differentiation on day 28. Scale bar, 50 μm (n = 5; mean ± SEM; ns, not significant, **p<0.01). **g** and **h.** TUNEL staining (g) and quantification of apoptotic cells (h) of each line at 28 days of differentiation. Scale bar, 50 μm (n = 5; mean ± SEM; ns, not significant, ***p<0.001). **i.** The cell morphology of A15-NPC-3 line transduced with the control and PSEN1-WT on 28 days of differentiation, respectively. Scale bar, 50 μm. **j.** Immunofluorescence staining of A15-NPC-3 line transduced with the control and PSEN1-WT on 28 days of differentiation, respectively, with antibodies against Nestin and MAP2. Scale bar, 50 μm. **k.** Quantification of percentages of MAP2+ neurons (n = 3; mean ± SEM; ns, not significant, ***p<0.001).

To verify the causal role of *PSEN1* mutations for the phenotype of premature neuronal differentiation, we over-expressed either wild type *PSEN1* (*PSEN1-WT*) or *PSEN1* with mutation of A246E (*PSEN1-A246E*) as well as an empty vector (control) into N-iPSC-1 cells (Supplementary Figure 3a) and induced all three types of iPSCs to neural differentiation as described above. The differentiation culture of NPCs from *PSEN1-A246E* transfected N-iPSC-1 at day 28 contained more cells with long neurites and had an obviously lower cell density than differentiation culture of NPCs from *PSEN1-WT* and vector transfected N-iPSC-1, in a way similar to differentiation culture of AD-NPCs (Figure 4a). Moreover, significantly lower percentage of Nestin^+^ NPCs and higher percentage of MAP2^+^ neurons were detected in *PSEN1-A246E*-transfected cells (Figure 4b and 4c). The number of Nestin^+^ cells was dramatically reduced in *PSEN1-A246E*-transfected cell cultures (Figure 4d). Furthermore, over-expression of *PSEN1-A246E* in N-iPSC-1 drastically reduced percentage of BrdU^+^ proliferative cells, while no alteration was found in *PSEN1-WT* transduced cells (Figure 4e and 4f). In addition, introduction of *PSEN1-A246E* into N-iPSC-1 increased the percentage of TUNEL^+^ apoptotic cells during differentiation (Figure 4g and 4h). Therefore, the introduction of mutated *PSEN1* into N-iPSCs produced similar early neural dysfunctions as seen in AD-iPSCs during neuronal differentiation. However, over-expression of *PSEN1-A246E* in N-iPSC-1 cells did not affect the maintenance of N-NPCs, as evidenced by normal survival, proliferation and expression of NPC markers in NPCs derived from the *PSEN1-A246E*-transfected N-iPSC-1 cells (Supplementary Figure 3b-3f).

Interestingly, over-expression of *PSEN1-WT* failed to prevent NPCs of A15-iPSC-3 from premature neuronal differentiation (Supplementary Figure 3g, Figure 4i-4k). The percentage of MAP2^+^ neurons was significantly higher in both vector transfected A15-NPC-3 (control) and *PSEN1-WT*-infected A15-NPC-3 cells than that in vector transfected N-NPC-1 controls (Figure 4j and 4k). The finding that WT PSEN1 could not correct the phenotypes in AD-NPCs during neuronal differentiation argues for a dominant nature of mutated PSEN1 in AD-NPCs.

### Knock down of PSEN1 expression in AD-NPCs prevents premature neuronal differentiation

**Figure 5:**
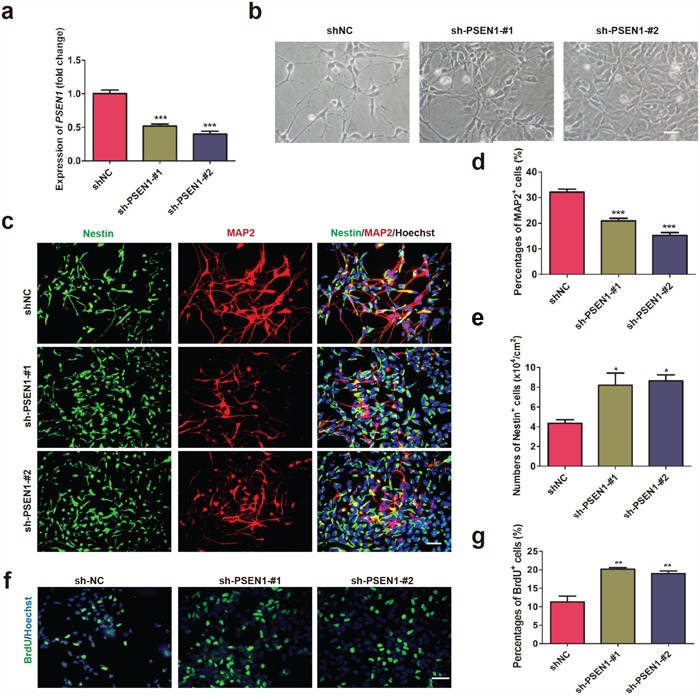
Knock down of PSEN1 in AD-NPCs rescues premature neuronal differentiation **a.** Transcript levels of PSEN1 in A15-NPC-3 transduced with a non-target sequence as negative control (shNC) and two shRNA sequences specific to PSEN1. The level of PSEN1 in A15-NPC-3 cells transduced with shNC was set as 1 (n = 3; mean ± SEM; ***p<0.001). **b.** Representative cell images at day 28 of differentiation in the cultures of each line. Scale bar, 50 μm. **c** to **e.** Nestin+ and MAP2+ cells of the three cell lines at day 28 of differentiation were detected by Immunofluorescence staining (c) and quantified (d and e). Scale bar, 50 μm (n = 3; mean ± SEM; *p< 0.05, ***p<0.001). **f** and **g.** Immunofluorescence staining of BrdU (f) and quantitative analysis (g) of BrdU+ cells on day 28 of neural differentiation. Scale bar, 50 μm (n = 3; mean ± SEM; **p<0.01).

Recently, Woodruff et al. demonstrated that *PSEN1* mutations disrupted γ-secretase activity and the mutation acted dominantly to execute toxic effects [28]. We hypothesized that a reduction in the level of endogenous mutated *PSEN1* expression in AD-NPCs might be able to correct premature neuronal differentiation of AD-NPCs. To this end, shRNA sequences specific to *PSEN1* were introduced into A15-NPC-3 (Figure 5a). Amazingly, knocking down the expression of *PSEN1* in AD-NPCs abrogated the phenotype of faster appearance of neurites observed in AD-NPCs at day 28 of differentiation (Figure 5b). Moreover, immunofluorescence staining results revealed that the knock down of *PSEN1* in AD-NPCs brought the percentage of MAP^+^ and Nestin^+^ cells closer to those in N-NPCs at day 28 of differentiation (Figure 5c-5e). Importantly, the number of Nestin^+^ NPCs and the percentage of proliferative cells were increased after *PSEN1* knock down (Figure 5e-5g). These results indicated that reduction of the mutant *PSEN1* expression in AD-NPCs could attenuate the abnormal neuronal differentiation.

### Gene expression profiles are altered in AD-NPCs during neuronal differentiation

**Figure 6:**
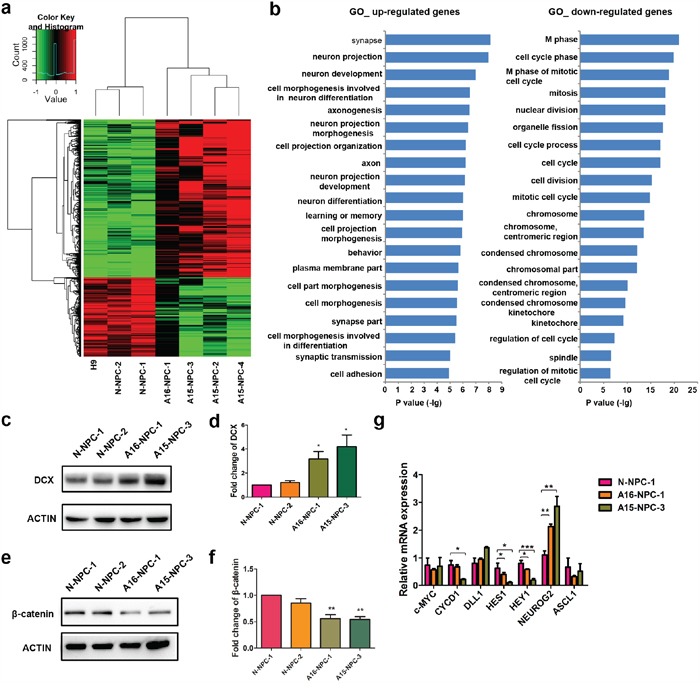
Genome-wide transcriptome analyses identify genes and signaling pathways potentially associated with abnormal neuronal differentiation in differentiating AD-NPCs **a.** Heat map of normalized expression levels for differentially expressed genes. Fold changes of each gene are indicated by the color key. **b.** Top 20 enriched GO terms analyzed by DAVID based on differentially expressed genes between AD-iPSCs (A16-iPSC-1, A15-iPSC-2, A15-iPSC-3 and A15-iPSC-4) and normal pluripotent stem cells (N-iPSC-1 and N-iPSC-2) at differentiation day 32. Up-regulated genes were defined by fold changes ≥ 2 and down-regulated genes were defined by fold changes ≤ 0.5. **c** and **d.** Protein levels of DCX in AD-NPCs at day 28 of neuronal differentiation were detected by western blotting (c) and quantified (d) by image J (n = 4; mean ± SEM; *p< 0.05). **e** and **f.** Protein levels of β-catenin in AD-NPCs on day 28 of neuronal differentiation were detected by western blotting (e) and quantified (f) by image J (n = 4; mean ± SEM; *p< 0.05). **g.** Transcript levelsfor several down-stream genes of Wnt and Notch pathways in differentiating N-NPCs and AD-NPCs at day 28 (n = 4; mean ± SEM; *p<0.05, **p<0.01, ***p<0.001).

To explore the molecular basis of premature neuronal differentiation in AD-NPCs, we compared the whole genome transcript profiling between four AD-NPCs lines and three normal control NPCs lines at day 32 of differentiation. Data analysis uncovered 430 up-regulated genes (at a change level ≥ 2 folds; p < 0.05) and 266 down-regulated genes (at a change level ≤ 0.5-fold; p < 0.05) (Figure 6a). Gene ontology (GO) analyses showed that up-regulated genes encode molecules associated with neuron development, neuron projection, neuron maturation, cell morphogenesis involved in neuron differentiation, which were in accordance with the abnormally enhanced neuronal differentiation observed in differentiated AD-NPCs. Down-regulated genes mainly encode the molecules involved in the regulation of cell cycle, nuclear division and condensed chromosomes, which might explain the reduction of cell proliferation and induction of cell apoptosis in differentiated AD-NPCs (Figure 6b). Among the up-regulated genes, there were previously reported AD associated genes, such as *HTR2A*, *ANK3*, *BDNF*, *IL18*, *LRP8*, *MAPT*, *PPARα*, *SLC18A3* and *VDR* (david.abcc.ncifcrf.gov). In addition, we screened for the up-stream transcription factors for the differentially expressed genes, which might control aberrant gene expression in AD-NPCs (Supplementary Figure 4a). The enriched transcriptional factors that ranked on the top were mostly related to neuronal differentiation, apoptosis and neuroprotection.

To further explore the molecular mechanisms underpinning the premature neuronal differentiation, we measured the protein level of doublecortin (DCX), a marker of immature newborn neurons [29]. We found that DCX level was markedly higher in AD-NPCs at day 28 of neuronal differentiation (Figure 6c and 6d) than that in N-NPCs. Furthermore, we identified β-catenin as one of the up-stream transcription factors of the differentially expressed genes (Supplementary Figure 4a). Western blot analyses revealed that its protein level decreased significantly in AD-NPCs at day 28 of neuronal differentiation (Figure 6e and 6f), suggesting that β-catenin might act as a repressor of neuronal differentiation. In addition, Notch signaling is known to play important roles in the maintenance of neural stem cells during development through controlling the transcription of its targets [30]. Interestingly, we found that the protein level of Notch intracellular domain (NICD), an intracellular effector of Notch signaling and a catalytic product of PSEN1 [31], was significantly decreased in AD-NPCs on day 28 of differentiation (Supplementary Figure 4b). In line with this finding, the transcript levels of Notch direct targets (*HES1* and *HEY1*) were significantly lower in differentiating AD-NPCs. In contrast, the level of proneural factor *NEUROG2*, which is transcriptionally repressed by HES1/HEY1 [32], was markedly higher in AD-NPCs than in N-NPCs during differentiation (Figure 6g).

## DISCUSSION

Recent investigations of modeling FAD with patient-specific iPSCs showed relatively late pathological manifestations of AD related to APP metabolism, Aβ accumulation or tau phosphorylation in neuronal or glia cells [15–19, 21, 22]. Previous studies have reported premature differentiation of NPCs in *PSEN1*^−/−^ mice [33]. It has been reported *in vitro* that NPCs isolated from 4 to 8-week-old *PSEN1ΔE9* transgenic mouse brains exhibited reduction in self-renewal capacity and premature neuronal differentiation [34]. These research findings in mouse models indicated that premature neuronal differentiation might occur during early neurogenesis in AD patients. In the current study, we focused on earlier cellular and molecular changes, which could be identified uniquely utilizing AD patient-specific iPSCs. Thus, we propose that the premature neuronal differentiation, along with enhanced apoptosis and reduced cell growth, would give rise to the progressive depletion of the NPC pool, which ultimately causes neuronal loss in the brain of AD patients. The proposal is consistent with the recent report that cell number change in the certain brain region is closely related to patients with AD or other types of dementia [10].

*PSEN1* mutants promote neurodegeneration and dementia without any increase in Aβ, suggesting the causal roles of *PSEN1* mutants in AD via an Aβ-independent manner [35–38]. *PSEN1* mutations might progressively impair neurogenesis, which is closely related to the memory deficits and cognitive decline in AD [39]. Neurogenesis occurs in the hippocampus throughout life. Alterations in notch and wnt signaling, mutations in *PSEN1* and *APP*, can impair the process of neurogenesis in AD [40]. Increase neurogensis or decreased neurogenesis happened in different stage of AD process [41]. Defection in maturation and reduction in the survival of newborn neurons, and compromised dendritic branching during neurogenesis are contributors to the cognition loss of AD [40]. Previous study found reduced hippocampal stem cells and higher DCX level in the dentate gyrus of AD patients’ brain [41]. We observed that the AD-NPCs grow slower than normal NPCs when transplanted to the hippocampus of immune-deficient mice (Supplementary Figure 5). Aβ accumulation has been considered as a late event in AD [42]. Here, we showed that the premature neuronal differentiation began earlier than the changes in Aβ_42_ and p-tau levels. Consistently, it has been reported that severely impaired proliferation and differentiation of NPCs occurrs preceding the onset of amyloid deposition, neurofibrillary tangles formation and memory impairment in either *APPswe/PSEN1ΔE9* or triple transgenic AD mice [43, 44]. Premature neuronal differentiation might gradually reduce NPC pool in the brain long before the emergence of AD clinical symptoms. Accumulating Aβ and p-tau in the brain of AD would further diminish NPC pool and accelerate memory deficit and cognitive decline. Thus, premature neuronal differentiation could be an important and unappreciated early pathogenic event in the pre-clinical stage of AD development, although more systemic investigations are needed.

The correction of neural functions in AD-NPCs through silencing the expression of mutated *PSEN1* is consistent with the finding in a recent study that some of *PSEN1* mutations are not simple loss of functional alleles with respective to the AD phenotype-associated biochemical pathway [28]. Moreover, that study suggests that γ-secretase activity is available in excess under the physiological conditions. Therefore, it is possible that certain FAD *PSEN1* mutations may gain functions to produce pathogenic effects. Thus, reduction in the expression level of *PSEN1* in AD-NPCs would possibly reduce the production of phenotype-associated molecules by the mutated allele, remaining the normal function by the wild-type allele. Supporting this notion, we found that over-expression of wild type *PSEN1* in AD-iPSCs is not able to correct the premature neuronal differentiation of AD-NPCs. Thus, simply increasing dosages of *PSEN1-WT* could not prevent or correct mutated *PSEN1*-induced NPCs dysfunctions. In contrast, a reduction in the endogenous level of mutant *PSEN1* is beneficial for FAD patients harboring *PSEN1* mutations (Figure 5c-5g).

Mutations in *PSEN1* could bring about alterations in multiple signaling pathways, such as Notch [31], syndecan 3 and N-cadherin [45], which in turn might participate in the development of neurodegeneration in AD. Borghese et. al have clearly demonstrated that inhibition of Notch signaling resulted in a marked acceleration of neuronal differentiation in human ESC-derived NPCs [46], which is consistent with our present finding. Moreover, it is reported that activated *HES1* and *HES5* expression induced by activation of Notch signaling could inhibit neuronal differentiation in mouse embryos, while *HES1* and *HES5* mutations would lead to premature neuronal differentiation in embryos [47, 48]. Thus, decreased levels of NICD proteins as well as *HES1* and *HEY1* transcripts observed in our AD-NPCs could be at least partially responsible for the premature neuronal differentiation. Additionally, β-catenin (*CNTTB1*) and its cofactor *LEF1* were identified as upstream transcription factors of the differentially expressed genes found between AD-NPCs and N-NPCs during differentiation. Hence, disturbed Wnt signaling might be also associated with the premature neuronal differentiation in AD-NPCs. Although iPSCs-based research in the present study provides a new perspective on AD pathogensis and treatment, we should be aware the complexity of genetic factors involved in the neurodegeneration of AD and the limitation of FAD-iPSCs modeling the sporadic AD. Further investigations of sporadic AD and other genetic defects caused FAD will help unfold the mystery of AD initiation and facilitate the development of more effective AD therapies.

## MATERIALS AND METHODS

### Cell culture and derivation of iPSC lines

Fibroblasts from a FAD patient carrying a *PSEN1* A246E mutation (A15, female, 56 years old) and another FAD patient carrying a *PSEN1* Ser169del mutation (A16, female, 45 years old) were obtained from the Xiangya Hospital with the approval of the Ethical Committee and informed consents of donors. Normal (N) fibroblasts from an unrelated subject were obtained from the Renji Hospital of Shanghai Jiao Tong University School of Medicine. Fibroblasts were cultured as previously described [49]. iPSC lines were established through infection of fibroblasts with retroviral vectors containing coding sequences of human *OCT4, SOX2, KLF4* and *C-MYC*. Procedures for retroviral production, iPSC derivation and characterization were the same as previously described [50]. iPSCs were cultured on CF1 or ICR MEF feeders for expansion in the human ESC medium (for detail please see Supplementary Information).

### NPC generation from iPSCs by RA treatment

NPCs were generated from iPSCs by RA treatment and neurosphere formation as previously described [25]. Briefly, iPSCs were cultured in the mTeSR1 medium containing 10 μM RA and 0.1 μM sodium butyrate (NaB) (Sigma, St. Louis, MO, USA) for 7 days. Afterwards, cells were digested with dispase (1 mg/ml, Millipore, Billerica, MA, USA) into small clumps containing 200 to 500 cells and cultured in suspension with the human ESC medium for 4 days to initiate formation of neurospheres, which were then cultured in the neurosphere (NSP) medium for 7 days. On day 18 of differentiation, neurospheres were digested into single NPCs, which could be either maintained as NPCs or further induced to neuronal differentiation.

The human ESC medium consisted of DMEM/F12 medium with 20% KSR, 1% GlutaMax-1, 1% non-essential amino acid (NEAA), 50 μM β-mercaptoethanol (ME), and 1% penicillin/streptomycin (P/S). The NSP medium consisted of DMEM/F12 medium containing 1% N2 supplement, 1% GlutaMax-1, 1% NEAA, 50 μM β-ME, 1% P/S, 8 μg/ml Heparin, 20 ng/ml bFGF, and 20 ng/ml Epidermal growth factor (EGF).

### Monolayer neural differentiation of iPSCs

N- and AD-iPSCs were firstly cultured in the mTeSR1 medium, and then the neural differentiation was proceeded as reported with a modification [26, 27]. Briefly, iPSCs were dissociated into single cells by accutase and replated onto Matrigel-coated dishes. When cells reached 95% full in the dish, the cultural medium was changed gradually from the KSR medium containing 500 ng/ml rmNoggin (R&D System, Minneapolis, MN, USA), 10 μM SB431542 (Tocris Bioscience, Bristol, UK) and sodium butyrate (STEMCELL Technologies, Vancouver, BC, Canada), to the N2 medium (DMEM/F12 supplemented with 1% L-Glutamine, 1% NEAA, 1% P/S and 1% N2 supplement) containing 500 ng/ml rmNoggin, 10 μM SB431542 and 0.1 mM sodium butyrate. At day 10 of induction, cells were passaged *en bloc* with dispase digestion and cultured in the N2B27 medium containing 20 ng/mL bFGF. Upon the appearance of polarized cells after being passaged for about one week, rosettes were mechanically picked up and dissociated into single cells by 0.05% trypsin. All later passages of NPCs were maintained in the N2B27 medium with 20 ng/ml of bFGF.

### NPC maintenance

NPCs were cultured on Matrigel-coated dishes in the NPC medium consisting of DMEM/F12 and neurobasal medium (1:1), 0.5% N2, 1% B27, 1% GlutaMax-1, 1% NEAA, 50 μM β-ME, 1% P/S, 10 ng/ml bFGF. NPCs differentiated in the NPC medium without bFGF for spontaneous neural differentiation into neuronal or glial cells.

### Directed neuronal differentiation

NPCs were re-plated on PLO/laminin-coated dishes or a 24-well plate at a density of 2 × 10^4^ cells/cm^2^ in the neuronal differentiation medium comprising the neurobasal medium, 1% N2, 2% B27, 1% GlutaMax-1, 1% NEAA, 50 μM β-ME, 1% P/S, 20 ng/ml brain-derived neurotrophic factor (BDNF), 20 ng/ml glial-derived neurotrophic factor (GDNF), 20 ng/ml ciliary neurotrophic factor (CNTF). All growth factors and cytokines were purchased from R&D (Minneapolis, MN, USA), media and supplements were from Invitrogen (Carlsbad, NY, USA).

### Statistical analysis

All data are presented as mean ± SEM values for 3 - 6 independent experiments and analyzed by the Student's *t* test or two-way ANOVA by Graph Pad Prism. When the value of *p* is < 0.05, the difference between two groups is considered as statistically significant.

## SUPPLEMENTARY MATERIALS DATA FIGURES AND TABLES





## Abbreviations

Aβ: β-amyloid
AD: Alzheimer's disease
AD-NPCs: AD-iPSC-derived NPCs
*APP*: amyloid precursor protein
*CNTTB1*: β-catenin
DCX: doublecortin
Ebs: embryoid bodies
FAD: familial AD
GO: Gene ontology
iPSCs: induced pluripotent stem cells
NICD: Notch intracellular domain
NPCs: neural progenitor cells
N-NPCs: N-iPSC-derived NPCs
*PSEN1*: presenilin-1
*PSEN2*: presenilin-2
p-tau: phosphorylated tau
*PSEN1-WT*: wild type *PSEN1*
*PSEN1-A246E*: *PSEN1* with mutation of A246E
