# Supplementary material for: Early pathogenic event of Alzheimer’s disease documented in iPSCs from patients with PSEN1 mutations
Source: Oncotarget. 2016 Dec 2;8(5):7900–13. doi: 10.18632/oncotarget.13776 (PMC5352369; doi:10.18632/oncotarget.13776)
Supplement: Supplementary file 1 [file oncotarget-08-7900-s001.pdf]

Early pathogenic event of Alzheimer's disease documented in iPSCs from patients with PSEN1 mutations

SUPPLEMENTARY DATA

SUPPLEMENTAL EXPERIMENTAL PROCEDURES

Immunofluorescence staining

Immunofluorescence staining was performed on fixed cells. Briefly, cells on glass coverslips were fixed in 4% paraformaldehyde (PFA) in PBS for 15 min at RT. The fixed cells were then blocked for 30 min in 3% goat serum in PBS supplemented with 0.1% Triton X-100 at RT followed by incubation with primary antibodies overnight at 4°C. After repeated wash for 3 times, cells were incubated with secondary antibodies conjugated with Alexa fluor-488/555 (Invitrogen) for 1 h at RT. The nuclei were finally stained with Hoechst for 3 min at RT before observation under a microscope (Olympus). Information for antibodies were as follows: OCT4 (1:100, homemade), SOX2 (1:500, homemade), TUJ1 (1:500, DAKO, Japan), AFP (1:500, Santa Cruz, Dallas, TX, USA), VIMENTIN (1:100, Millipore), SOX17 (1:100, R&D systems), FLK1 (1:100, Santa Cruz), Nestin (1:800, Millipore), MAP2 (1:500, Santa Cruz), MAP2 (1:1000, Millipore), NeuN (1:500, Millipore), SYP (1:10, Millipore), Alexa Fluor-488/555 (1:400, Invitrogen).

For quantitative analysis of immunofluorescence staining data, cells were plated on coverslips in a 24-well plate. At each day point (from differentiation days 21 to 36), one coverslip was taken out for immunofluorescence staining. Images from 3 to 6 fields of view were randomly taken under a microscope. The percentage of neurons was calculated by the number of $MAP2^+$ cells versus the number of $Hoechst^+$ cells. The average percentage of neurons was calculated from 4 - 6 images for each day point. The percentage of each day point for each cell line was determined by data from 4 - 6 independent experiments.

RT-PCR and quantitative RT-PCR (RT-qPCR)

Total RNA was extracted from cells using TRIzol (Invitrogen) and was transcribed into cDNA using oligo$(dT)_{15}$ and Rever-Tra Ace reverse transcriptase (Toyobo, Osaka, Japan). PCR reactions were carried out by mixing 1 µl of cDNA template, 250 nM of each primer, 200 mM dNTP mixture and 1 U of Taq DNA polymerase in a volume of 20 µl. For RT-qPCR, *GAPDH* was used as an inner control. Amplification data were collected using the ABI PRISM 7900 and analyzed using the Sequence Detection System 2.3 software. Primer information is provided in Supplementary Table 2.

Western blot analysis

Total proteins of cells at day 28 of differentiation were extracted by the radioimmunoprecipitation assay (RIPA) lysis buffer (Beyotime, Jiangsu, China) and western blotting was performed as described previously [1]. Antibodies used in this analysis include DCX (1:2,000; Cell Signaling, Danvers, MA, USA), Notch1 (1:1,000; Cell Signaling), NICD (1:400; Millipore), p-tau (1:1000; Millipore).

Bisulfite sequencing

Genomic DNA from patient fibroblast cells, human ESCs (SHhES3, which is previously established in our labratory) and AD-iPSCs was restricted with E*co*RV and treated with sodium bisulfite as previously described [2]. Treated DNA was subjected to nested PCR. Primer information is provided in Supplementary Table 2. The PCR products were cloned into T-vectors (Promega, Madison, WI, USA) and individually sequenced.

Teratoma formation

iPSCs from a 60 mm dish of each iPSC line at about 70% - 80% confluence were harvested and injected intramuscularly into immune-deficient NOD-SCID mice together with Matrigel and Y27632. Six to ten weeks later, teratomas were harvested and processed with hematoxylin and eosin (H&E) staining. All animal procedures were carried out according to the ethical guidelines of the Ruijin Hospital, Shanghai JiaoTong University School of Medicine.

BrdU incorporation

Ten µM BrdU (Sigma, St. Louis, USA) was incorporated into cells for 30 min in an incubator. Cells were then fixed with 4% PFA for 10 min, followed by 2 M HCl treatment for 30 min at 37°C. Subsequently, cells were stained with the BrdU antibody (1:400, Cell Signaling, Danvers, MA, USA) as described above.

TUNEL staining

Apoptotic cells were detected by the TUNEL staining with an *in situ* cell death detection kit (Roche).

Aβ ELISA

Four ml of cell culture medium was freeze-dried by a freeze drying equipment (Labconco Corporation, Kansa City, MO, USA) and then suspended in 200 μl buffer for ELISA. The secreted $A\beta_{42}$ and $A\beta_{40}$ were detected by a high sensitive ELISA Kit (WAKO, Chuo-ku, Osaka, Japan).

Virus packaging and stably-infected cell line screening

PMXS retroviruses containing coding sequences of human *OCT4*, *SOX2*, *KLF4* and *C-MYC* were used for iPSC generation. PLVX lentiviral vectors containing coding sequences of human *PSEN1-WT* or *PSEN1-A246E* were used for overexpression study. And pLKO.1 lentiviral vector was employed to knock-down human *PSEN1*. The shRNA oligos targeting *PSEN1* were 5'-GGGACGAGGTCAAGGAGATAT-3' and 5'-GCCTTTGGCAATTCTTCTTCT-3', respectively. Viruses were packaged and stably-infected cell lines were screened as described previously [3].

Electrophysiology

Whole-cell recording was performed with cultured neurons as described previously [4].

Microarray analysis

Experiments were carried out with Affymetrix U133 plus 2.0 gene chips at the Shanghai Biotechnology Corp. For each line of cells, one sample was prepared according to Affymetrix Technical Manual. To obtain differentially expressed genes between AD-NPCs and normal NPCs, mRNA samples from 4 lines of AD-NPCs and 2 lines of normal NPCs were prepared at day 32 of neural differentiation. Moreover, differentially expressed genes in our microarray results were used for upstream transcriptional factor identification based on reported Chip-seq data or motif prediction using *Expression2Kinases* as previously described [5]. For gene expression profiles of undifferentiated human pluripotent stem cells, raw data normalization and scatter plots analysis were performed as previously described [6].

NPC transplantation

The NPCs derived from iPSCs were stable transfected with pCDH-EGFP plasmid under the control of the EF1α promoter using Fugene HD kit (Roche) and were cultured in the NPC medium with 1 mg/ml puromycin. We prepared NPCs from 5 iPSC lines including N-iPSC-1, N-iPSC-2, A15-iPSC-2, A15-iPSC-3 and A16-iPSC-1, respectively. Before transplantation, the immune-deficient null mice (8 weeks old) were anesthetized with 10% chloral hydrate, and then received 2 μl of NPC suspension (~1 × 10^5 cells) unilaterally to the dentate gyrus (DG) region of the hippocampus. Six weeks after transplantation, mice were sacrificed for serial section of the hippocampus for histological study. hNA positive cells were counted in a one-in-four series, 20 sections in total. Immunofluorescence staining

REFERENCES

1. Li L, Sun L, Gao F, Jiang J, Yang Y, Li C, et al. Stk40 links the pluripotency factor Oct4 to the Erk/MAPK pathway and controls extraembryonic endoderm differentiation. Proceedings of the National Academy of Sciences of the United States of America. 2010;107:1402-7.
2. Fong YW, Cattoglio C, Yamaguchi T, Tjian R. Transcriptional regulation by coactivators in embryonic stem cells. Trends Cell Biol. 2012;22:292-8.
3. Yang J, Tang Y, Liu H, Guo F, Ni J, Le WD. Suppression of histone deacetylation promotes the differentiation of human pluripotent stem cells towards neural progenitor cells. Bmc Biol. 2014;12:Epub.
4. Xu C, Zhao MX, Poo MM, Zhang XH. GABA(B) receptor activation mediates frequency-dependent plasticity of developing GABAergic synapses. Nature neuroscience. 2008;11:1410-8.
5. Chen EY, Xu HL, Gordonov S, Lim MP, Perkins MH, Ma'ayan A. Expression2Kinases: mRNA profiling linked to multiple upstream regulatory layers. Bioinformatics. 2012;28:105-11.
6. Li C, Yu H, Ma Y, Shi G, Jiang J, Gu J, et al. Germline-competent mouse-induced pluripotent stem cell lines generated on human fibroblasts without exogenous leukemia inhibitory factor. PloS one. 2009;4:e6724.

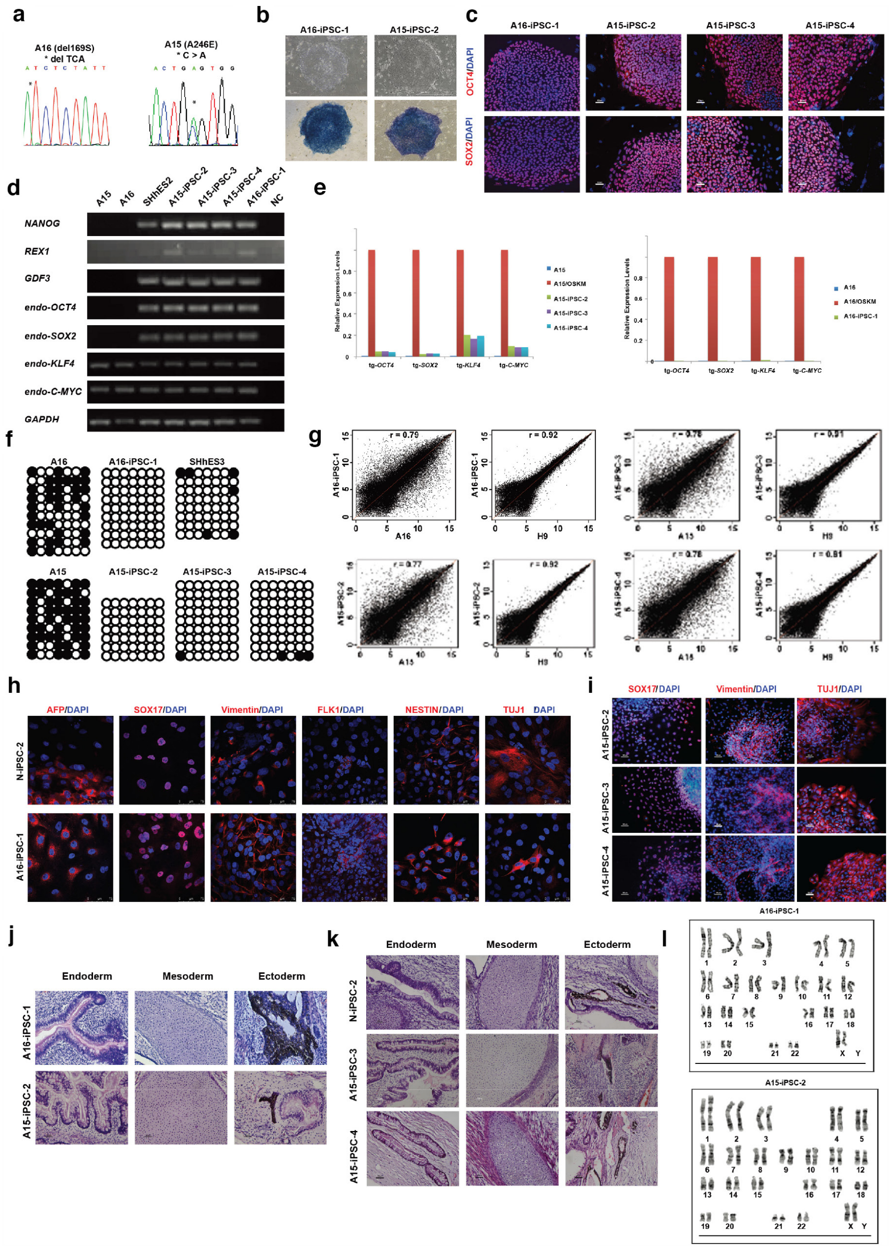

Supplementary Figure 1: Characterization of established normal and AD-iPSC lines. a. Genomic DNA sequencing showing heterozygous *PSEN1* mutations of 169 serine deletion in A16 fibroblast cells (S169del) and 246 missense mutation in A15 fibroblast cells (A246E), respectively, from FAD patients. **b.** Morphology and alkaline phosphatase staining of AD-iPSCs. Scale bars, 100 μm. **c.** Immunofluorescence staining of AD-iPSCs using antibodies against OCT4 and SOX2. Scale bars, 50 μm. **d.** Transcript levels of endogenously expressed pluripotency-associated genes in various AD-iPSC lines and one human ESC line determined by RT-PCR assays. 'NC' denotes a negative control without the reverse transcriptase. **e.** Transcript levels of 4 transgenic factors *OCT4*, *SOX2*, *KLF4* and *C-MYC* in AD-iPSCs determined by real-time RT-qPCR assays. The level of each gene in A16 or A15 cells infected with retroviruses encoding *OCT4*, *SOX2*, *KLF4* and *C-MYC* for 5 days (A16/OSKM or A15/OSKM) was set as 1. **f.** Bisulfite PCR and sequencing analysis of *OCT4* promoter methylation in AD-iPSCs. Open and closed circles represent unmethylated and methylated CpG sites, respectively. **g.** Global gene expression profiles of AD-iPSCs were compared with those of starting fibroblast cells, by a scatter plots analysis; the 'r' represents the correlation efficient. **h.** Immunofluorescence staining of embryoid bodies (EBs) generated from N-iPSC-2 and A16-iPSC-1 using antibodies against AFP and SOX17 (endoderm), Vimentin and FLK1 (mesoderm), Nestin and TUJ1 (ectoderm). Scale bars, 75 μm. **i.** Immunofluorescence staining of EBs formed by A15-iPSC-2, -3 and -4 with antibodies against SOX17 (endoderm), Vimentin (mesoderm) and TUJ1 (ectoderm). Scale bars, 50 μm. **j** and **k.** H&E staining of teratoma sections generated from AD-iPSCs, including goblet cells (endoderm), cartilage (mesoderm), and pigment cells (ectoderm). Scale bars, 50 μm. **l.** G-banding analysis of A16-iPSC-1 with a normal 46, XX karyotype and A15-iPSC-2 with a 46, XX (t (1; 9) (p32; q11)) karyotype.

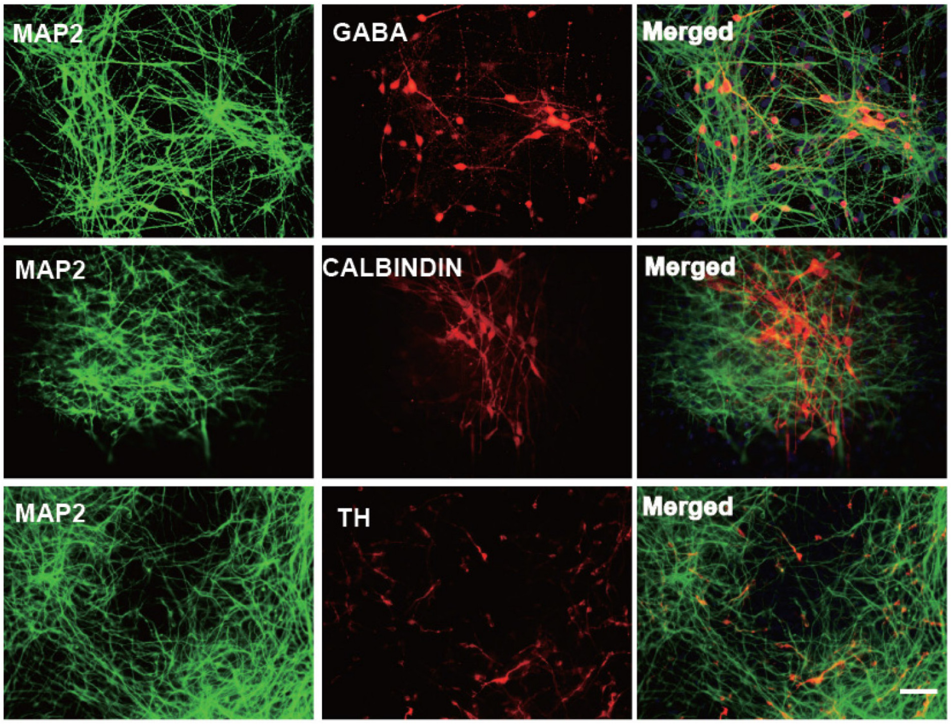

Supplementary Figure 2: Neurons (MAP2+, green) derived from NPCs were detected. Different neuronal subtypes including GABA neurons (from A15-iPSC-2), CALBINDIN neurons (from A15-iPSC-2) and TH neurons (from N-iPSC-1) were detected after 30 days of differentiation. Scale bar, 50µm.

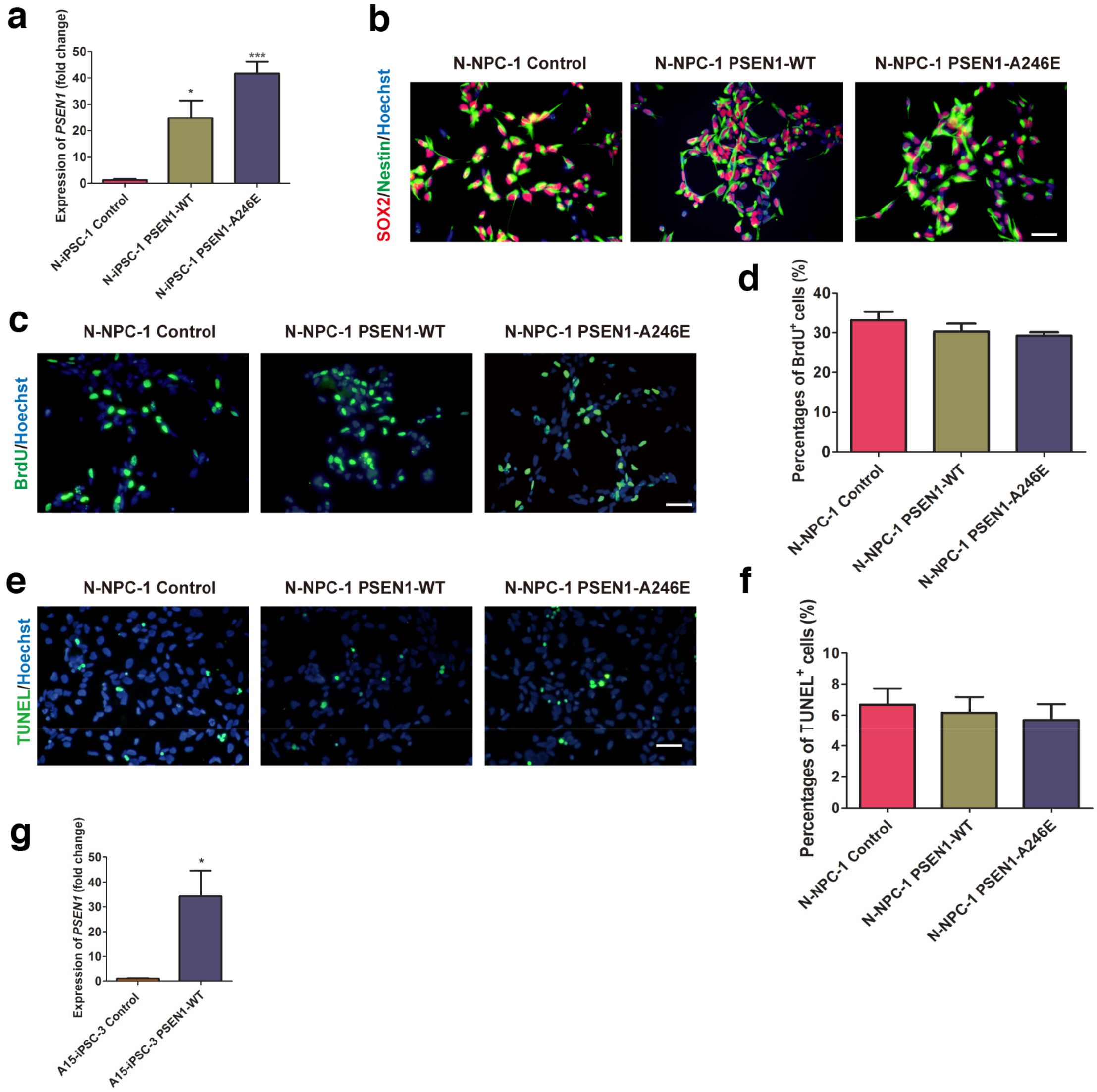

Supplementary Figure 3: Characterization of NPCs derived from N-iPSC-1 Control, N-iPSC-1 *PSEN1-WT* and N-iPSC-1 *PSEN1-A246E* as well as neurons derived from A15-iPSC-3 Infected with *PSEN1-WT* (related to Figure 3). **a.** Expression levels of *PSEN1* in the N-iPSC-1 infected with Control, *PSEN1-WT*, and *PSEN1-A246E* determined by real-time RT-qPCR assays. The expression level of *PSEN1* in N-iPSC-1 cells infected with a control vector was set as 1. The error bars indicate SEM. n=3; *, p <0.05; ***, p <0.001. **b.** Immunofluorescence staining of NPCs from each cell line with antibodies against SOX2 and Nestin. The nucleus was stained by Hoechst. Scale bar, 50 μm. **c** and **d.** Immunofluorescence staining of NPCs from each cell line with BrdU antibody and statistic results of BrdU incorporation assays. Scale bar, 50 μm. Error bars indicate SEM. n=3. **e** and **f.** The apoptosis of each NPC line was measured by TUNEL staining. Scale bar, 50 μm. Error bars indicate SEM. n=3. **g.** Examination of the expression levels of *PSEN1* in the A15-NPC-3 infected with a control vector or *PSEN1-WT* construct. The expression level of *PSEN1* in A15-NPC-3 cells infected with a control vector was set as 1. Error bars indicate SEM. n=3; *, $p < 0.05$.

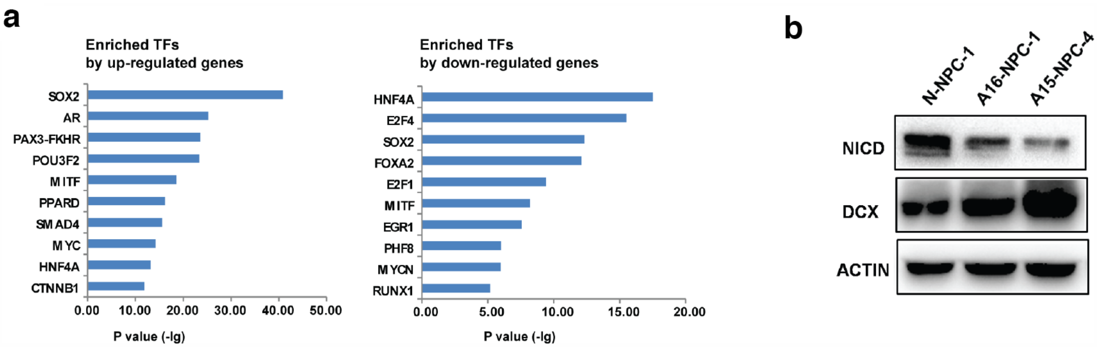

Supplementary Figure 4: The genome-wide transcriptome analysis of iPSC-NPCs during neuronal differentiation (related to Figure 5). a. Transcription factors with binding motifs enriched in the regulatory sequences of the differentially expressed genes determined by the X2K based on published ChIP-seq data. **b.** Typical western blotting of NICD and DCX for each cell line on day 28 of neuronal differentiation. Four independent experiments were conducted.

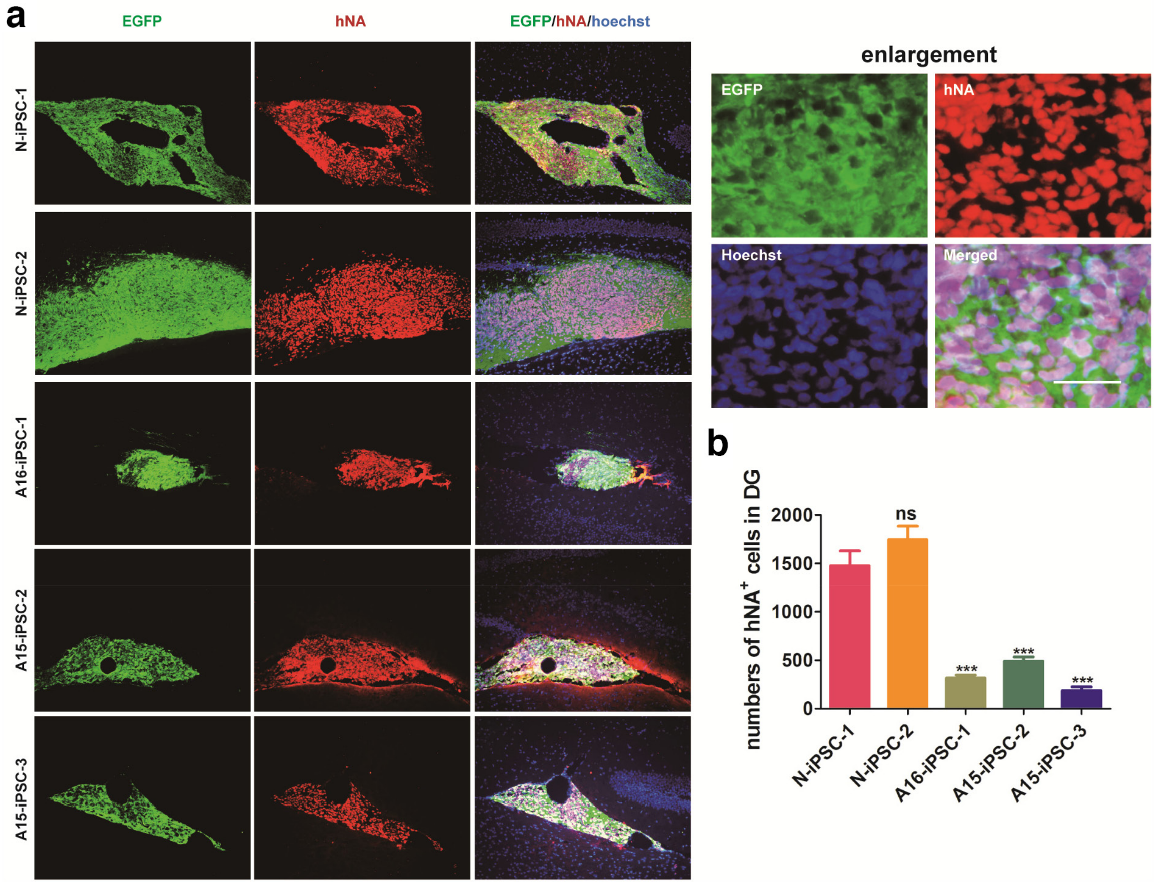

Supplementary Figure 5: Defects of AD iPSC-NPCs after transplantation into the hippocampus of immune-deficient mice. a. Engrafted cells at DG regions were detected by antibodies against EGFP and hNA. Scale bar, 50 μm **b.** The average numbers of hNA^+ cells were counted from a one-in-four serial sections of each mice. Data are shown as mean ± SEM. Ns, $p > 0.05$; ***, $p < 0.0001$.

Supplementary Table 1: Results of STR analysis

	D13S317	D16S539	TH01	D7S820	CSF1PO	D5S818	TP0X
A15-iPSC-4	185.54	146.16	152.09	212.9	305.5	142.92	231.78
	189.68	158.78	171.31	217.08	313.73	147.12	*
A15-iPSC-3	185.58	146.16	151.99	212.97	306.48	*	231.52
	189.61	158.67	171.22	217.17	314.67	147.64	*
A15-iPSC-2	185.75	146.08	151.79	213.2	305.47	*	231.52
	189.68	158.76	171.06	217.37	313.67	147.39	*
A15	185.92	146.19	151.87	212.86	306.53	142.56	231.78
	189.93	158.87	171.08	217.31	314.74	147.04	*
SHhES2	173.36	154.58	156.05	204.75	305.31	147.5	231.61
	189.68	162.71	168.11	217.24	309.4	155.86	239.82
N-iPSC-1	173.45	150.71	168.17	221.33	*	147.48	231.65
	181.59	158.84	171.22	*	313.48	151.81	243.87

* Undetermined.

Supplementary Table 2: The primer sequences used in this study

See Supplementary File 1
