# Supplementary material for: Early pathogenic event of Alzheimer’s disease documented in iPSCs from patients with PSEN1 mutations
Source: Oncotarget. 2016 Dec 2;8(5):7900–13. doi: 10.18632/oncotarget.13776 (PMC5352369; doi:10.18632/oncotarget.13776)
Supplement: Supplementary file 2 [file oncotarget-08-7900-s002.doc]

**Supplemental Table 2** The primer sequences used in this study.

| Gene | Forward primer sequences 5'-3' |
| --- | --- |
|  | Reverse primer sequences 5'-3' |
|  | RT-qPCR |
| OCT4 A | CTCCTGGAGGGCCAGGAATC |
|  | CCACATCGGCCTGTGTATAT |
| ed-SOX2 | GGGAAATGGGAGGGGTGCAAAAGAGG |
|  | TTGCGTGAGTGT GGATGGGATTGGTG |
| ed-KLF4 | ACGATCGTGGCCCCGGAAAAGGACC |
|  | TGATTGTAGTGCTTTCTGGCTGGGCTCC |
| NANOG | CAGCCCCGAπCTTCCACCAGTCCC |
|  | CGGAAGATTCCCAGTCGGGTTCACC |
| REX1 | CAGATCCTAAACAGCTCGCAGAAT |
|  | GCGTACGCAAATTAAAGTCCAGA |
| GDF3 | CTTATGCTACGTAAAGGAGCTGGG |
|  | GTGCCAACCCAGGTCCCGGAAGTT |
| GAPDH | TCCACCCATGGCAAATTCC |
|  | TCGCCCCACTTGAπTTGG |
| tg-hOCT4 | CCCCAGGGCCCCATTTTGGTACC |
|  | TTATCGTCGACCACTGTGCTGCTG |
| tg-hSOX2 | GGCACCCCTGGCATGGCTCTTGGCTC |
|  | TTATCGTCGACCACTGTGCTGCTG |
| tg-hKLF4 | ACGATCGTGGCCCCGGAAAAGGACC |
|  | TTATCGTCGACCACTGTGCTGCTG |
| tg-hC-MYC | CAACCGA AAATGCACCAGCCCCAG |
|  | TTATCGTCGACCACTGTGCTGCTG |
| 18srRNA | CAGCCACCCGAGATTGAGCA |
|  | TAGTAGCGACGGGCGGTGTG |
| C-MYC | CGAGAGGACCCGTGGATGCAGAG |
|  | TTGAGGGGCATCGTCGCGGGAGGCTG |
| CYCD1 | AACTACCTGGACCGCTTCCT |
|  | CCACTTGAGCTTGTTCACCA |
| HES1 | AAGAAAGATAGCTCGCGGCA |
|  | TACTTCCCCAGCACACTTGG |
| DLL1 | ACCTCGCAACAGAAAACCCA |
|  | GTGTTCGTCACACACGAAGC |
| HEY1 | AGCCGAGATCCTGCAGATGA |
|  | GCCGTATGCAGCATTTTCAG |
| Neurog2 | CTTGCAGCTTTCACGCCG |
|  | TGACGAACATCTTAGTTGGCTCT |
| ASCL1 | GGAGCTTCTCGACTTCACCA |
|  | AACGCCACTGACAAGAAAGC |
| mGapdh | GTCGTGGAGTCTACTGGTGTC |
|  | GAGCCCTTCCACAATGCCAAA |
| mNestin | TGAGGGTCAGGTGGTTCTG |
|  | AGAGCAGGGAGGGACATTC |
| mHes1 | GAGAAGAGGCGAAGGGCAAGA |
|  | TTCCGGAGGTGCTTCACAGTC |
| mHes5 | GATATTTGTAGAGTCGGGGGTT |
|  | GCTGAGTGCTTTCCTATGAGGA |
| mHey1 | GCCCTGGCTATGGACTATCG |
|  | CGCTGGGATGCGTAGTTGT |
| mNotch1 | CCCTTGCTCTGCCTAACGC |
|  | GGAGTCCTGGCATCGTTGG |
| mNeurog2 | AACTCCACGTCCCCATACAG |
|  | GAGGCGCATAACGATGCTTCT |
| mDcx | GGCCAAGAAGGTACGTTTCTAC |
|  | AGCAACGCATCAAAACTACGAA |
|  | Bisulfate sequencing |
| hOCT4-Outside | GTTAAGGTTAGTGGGTGGGATT |
|  | ATCACCTCCACCACCTAAAAA |
| hOCT4-Inside | AGAGAGGGGTTGAGTAGTTTTTT |
|  | ACCTCCACCACCTAAAAAAAAC |
|  | PSEN1 sequencing |
| PSEN1 | CCTATAACGTTGCTGTGGACTACATTACTG |
|  | CATATACTGAAATCACAGCCAAGATGAGCC |

ed: endogenous genes

tg: transgenic genes

m: mouse

h: human
